# Supplementary material for: Economic evaluation of trastuzumab in HER2-positive early breast cancer in Indonesia: A cost-effectiveness analysis
Source: PLoS One. 2024 May 24;19(5):e0304483. doi: 10.1371/journal.pone.0304483 (PMC11125485; doi:10.1371/journal.pone.0304483)
Supplement: S1 File — (DOCX) [file pone.0304483.s001.docx]

Supplementary Material

**Economic evaluation of trastuzumab in HER2-positive early breast cancer in Indonesia: a cost-effectiveness analysis**

Sudewi Mukaromah Khoirunnisa^1,2*^, Fithria Dyah Ayu Suryanegara^1,3^, Didik Setiawan^4,5^, Maarten Jacobus Postma^1,6,7,8^, Lisa Aniek de Jong^1^

^1^Department of Health Sciences, University of Groningen, University Medical Center Groningen, Groningen, the Netherlands

^2^Department of Pharmacy, Institut Teknologi Sumatera, Lampung Selatan, Indonesia

^3^Department of Pharmacy, Universitas Islam Indonesia, 55584, Yogyakarta, Indonesia

^4^Faculty of Pharmacy, Universitas Muhammadiyah Purwokerto, Banyumas, Indonesia

^5^Centre for Health Economic Studies, Universitas Muhammadiyah Purwokerto, Banyumas, Indonesia

^6^Department of Economics, Econometrics and Finance, University of Groningen, Faculty of Economics & Business, Groningen, the Netherlands

^7^Department of Pharmacology and Therapy, Faculty of Medicine, Universitas Airlangga, Surabaya, Indonesia

^8^Centre of Excellence in Higher Education for Pharmaceutical Care Innovation, Universitas Padjadjaran, Bandung, Indonesia

***Corresponding author:**

Sudewi Mukaromah Khoirunnisa, MSc, Apt

E-mail: [s.m.khoirunnisa@rug.nl](mailto:s.m.khoirunnisa@rug.nl), [sudewi.mukaromah@fa.itera.ac.id](mailto:sudewi.mukaromah@fa.itera.ac.id)

Phone: (+31) 627862661

We requested sample data from BPJS specific to breast cancer patients with The International Classification of Diseases (ICD) version 10 code C50, consisting of the following variables: patient ID, visit date, discharge date, province, healthcare type, INACBG code, procedures received by patients, and costs.

For information on requesting data from BPJS Kesehatan, we accessed the JKN Portal at <https://data.bpjs-kesehatan.go.id/> and followed these steps:


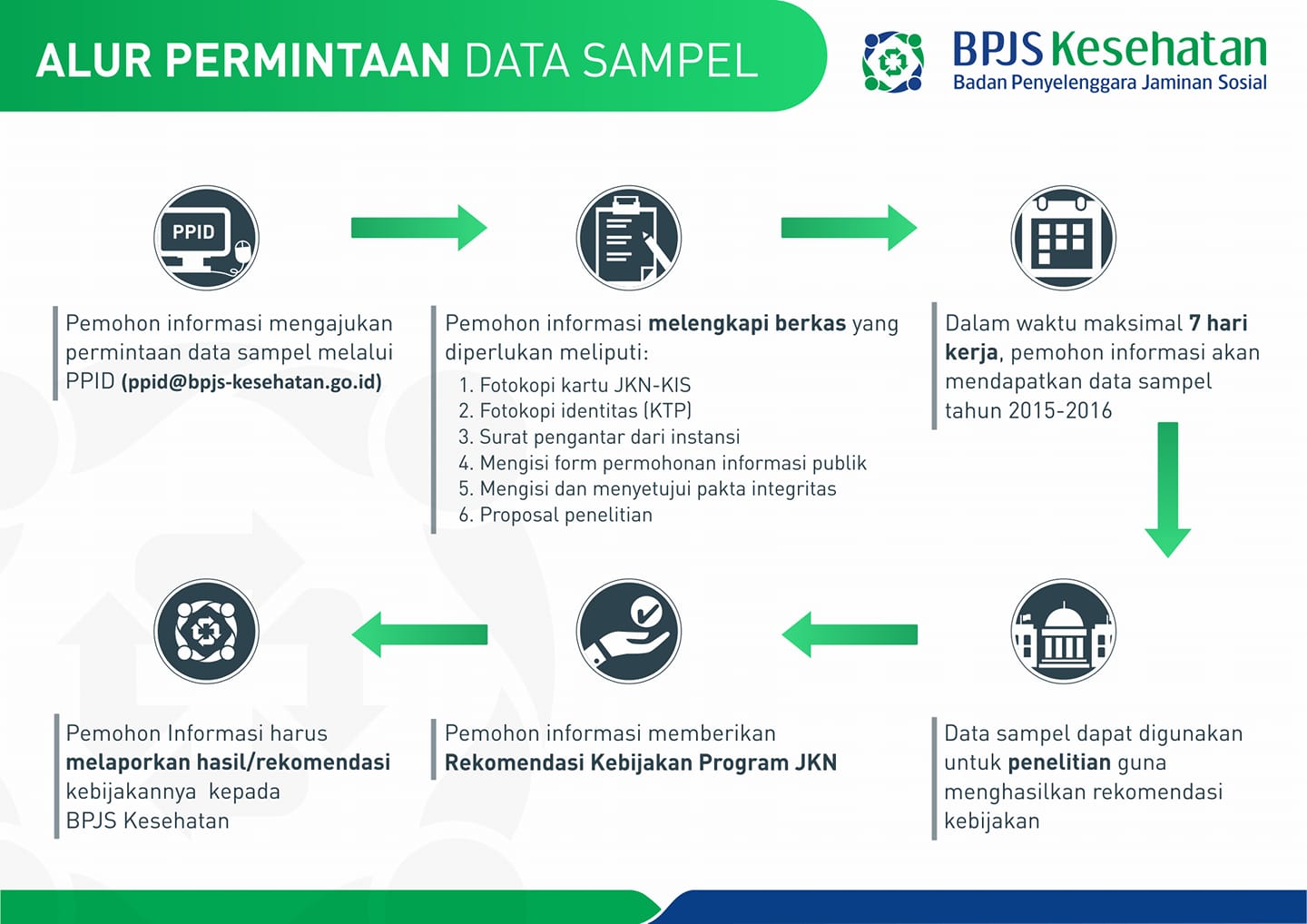


Figure 1. Steps in requesting data sampel from BPJS (source: <https://data.bpjs-kesehatan.go.id/>).
