# Supplementary material for: Economic evaluation of trastuzumab in HER2-positive early breast cancer in Indonesia: A cost-effectiveness analysis
Source: PLoS One. 2024 May 24;19(5):e0304483. doi: 10.1371/journal.pone.0304483 (PMC11125485; doi:10.1371/journal.pone.0304483)
Supplement: Attachment — Submitted filename: PLOS ONE comments.pdf [file pone.0304483.s002.pdf]

The manuscript is very interesting and well written, although I believe these comments could improve your manuscript.

The abstract and introduction are interesting and well written.

Materials and Methods

Table 1 and Table 2; Hazard ratio overall survival, Hazard ratio Disease-free survival, and Hazard ratio for distant metastasis in table 1 from HERA trial, and table 2 from the joint analysis of NSABP B-31 and NCCTG177 N98313, why did you use table 1 inputs for base case with ICER 6,842 per QALY then use table 2 input for total population included in the joint analysis with ICER was US$5,292 per QALY, however you could use the table 2 inputs for both base case and Scenario analysis (age subgroup), please clarify the value of using both data as it could be confusing for the reader. Note extracting HR for general population from a meta-analysis for the general population will give you more robust results instead of using 2 different RCT respecting to the recommendation of evidence ranking in health economics (less confusing and more consistent), there are a meta-analysis 2021 covering this topic

Line 195 “per-capita GDP” please add the value of per capita GDP in table 1 and illustrate per capita GDP for which year.

Line 241 “For the total population included in the joint analysis, the ICER was US$5,292 per QALY.” It is not clear how this ICER was calculated (depends on which HR)

Line 323 HERA trial you need to add the reference here.

Line 158 “For estimating costs per health states, the proportion of resources used for each health states was utilized to finally calculate the total cost annually” as you depended on procedures costs from another country (India), it would be better to mention that in the limitation, as economic evaluations depend mainly on the costs in your country to be freely compare the ICER to your WTP threshold.

Results are well interpreted , although it is recommended to improve the resolution of figure 2,3,4,5,6.

The discussion is well written.

Very important topic but you need to clarify a few points to be more reliable and reproducible
